# Effects of inhaled iloprost on right ventricular contractility, right ventriculo-vascular coupling and ventricular interdependence: a randomized placebo-controlled trial in an experimental model of acute pulmonary hypertension

**DOI:** 10.1186/cc7005

**Published:** 2008-09-10

**Authors:** Steffen Rex, Carlo Missant, Piet Claus, Wolfgang Buhre, Patrick F Wouters

**Affiliations:** 1 Department of Acute Medical Sciences, Centre for Experimental Anaesthesiology, Emergency and Intensive Care Medicine, Catholic University Leuven, Minderbroedersstraat, 3000 Leuven, Belgium; 2 Department of Anaesthesiology and Department of Intensive Care Medicine, University Hospital of the Rheinisch-Westfälische Technische Hochschule Aachen, Pauwelsstraße, 52074 Aachen, Germany; 3 Department of Cardiovascular Diseases, Division of Imaging and Cardiovascular Dynamics, Catholic University Leuven, UZ Herestraat, 3000 Leuven, Belgium; 4 Department of Anaesthesia and Intensive Care Medicine, Hospital Køln-Merheim, University of Witten-Herdecke, Ostmerheimer Straße, 51109 Køln, Germany; 5 Department of Anaesthesia, University Hospitals Ghent, De Pintelaan, 9000 Ghent, Belgium

## Abstract

**Introduction:**

Prostacyclin inhalation is increasingly used to treat acute pulmonary hypertension and right ventricular failure, although its pharmacodynamic properties remain controversial. Prostacyclins not only affect vasomotor tone but may also have cAMP-mediated positive inotropic effects and modulate autonomic nervous system tone. We studied the role of these different mechanisms in the overall haemodynamic effects produced by iloprost inhalation in an experimental model of acute pulmonary hypertension.

**Methods:**

In this prospective, randomized, placebo-controlled animal study, twenty-six pigs (mean weight 35 ± 2 kg) were instrumented with biventricular conductance catheters, a pulmonary artery flow probe and a high-fidelity pulmonary artery pressure catheter. The effects of inhaled iloprost (50 μg) were studied in the following groups: animals with acute hypoxia-induced pulmonary hypertension, and healthy animals with and without blockade of the autonomic nervous system.

**Results:**

During pulmonary hypertension, inhalation of iloprost resulted in a 51% increase in cardiac output compared with placebo (5.6 ± 0.7 versus 3.7 ± 0.8 l/minute; *P *= 0.0013), a selective reduction in right ventricular afterload (effective pulmonary arterial elastance: 0.6 ± 0.3 versus 1.2 ± 0.5 mmHg/ml; *P *= 0.0005) and a significant increase in left ventricular end-diastolic volume (91 ± 12 versus 70 ± 20 ml; *P *= 0.006). Interestingly, right ventricular contractility was reduced after iloprost-treatment (slope of preload recruitable stroke work: 2.2 ± 0.5 versus 3.4 ± 0.8 mWatt·s/ml; *P *= 0.0002), whereas ventriculo-vascular coupling remained essentially preserved (ratio of right ventricular end-systolic elastance to effective pulmonary arterial elastance: 0.97 ± 0.33 versus 1.03 ± 0.15). In healthy animals, inhaled iloprost had only minimal haemodynamic effects and produced no direct effects on myocardial contractility, even after pharmacological blockade of the autonomic nervous system.

**Conclusions:**

In animals with acute pulmonary hypertension, inhaled iloprost improved global haemodynamics primarily via selective pulmonary vasodilatation and restoration of left ventricular preload. The reduction in right ventricular afterload is associated with a paradoxical decrease in right ventricular contractility. Our data suggest that this reflects an indirect mechanism by which ventriculo-vascular coupling is maintained at the lowest possible energetic cost. We found no evidence for a direct negative inotropic effect of iloprost.

## Introduction

Because of its marked pulmonary vasodilating effects, ease of administration and lack of toxicity, intermittent nebulization of iloprost has become an established therapy in chronic pulmonary hypertension (PHT) [1] and is increasingly being used to treat postcardiotomy right ventricular (RV) dysfunction [2-4].

There is evidence that ventricular afterload reduction may not be the sole mechanism by which iloprost, the stable carbacyclin derivative of prostacyclin (prostaglandin I_2 _[PGI_2_]), improves cardiac performance in PHT. PGI_2 _stimulates the intracellular synthesis of cAMP [5], and was therefore postulated to have direct positive inotropic effects [6]. However, animal studies have produced conflicting results, showing positive [7], negative [8] or no inotropic effects [9] of PGI_2 _infusion in various models. A clinical study demonstrated that in PHT, inhaled iloprost increases cardiac output more than nitric oxide [10]. Prostanoids are also known to modify the autonomic nervous system (ANS) both indirectly (through hypotension-induced baroreflex activation) and via direct receptor-mediated effects on sympathetic and parasympathetic nerves [11-14]. It is reasonable to expect that these diverse pharmacodynamic actions contribute to the haemodynamic effects even of inhaled iloprost, because inhalation of a single clinical dose produces significant spill-over into the systemic circulation for at least 20 minutes, with up to 76% of the aerosolized iloprost appearing intravascularly [15,16]. In the present study we aimed to elucidate the precise mechanism(s) through which inhaled iloprost affects the cardiovascular system. We used the current 'gold standard' methods to quantify biventricular contractile performance and cardiac loading conditions in an experimental model for acute PHT as well as in healthy animals with intact and pharmacologically blocked ANS.

## Materials and methods

This investigation conforms to the *Guide for the Care and Use of Laboratory Animals*, published by the US National Institutes of Health (Publication No. 85-23, revised 1996) [17] and was approved by the ethics committee of the Katholieke Universiteit Leuven, Belgium.

### Instrumentation

Twenty-six pigs (mean weight 35 ± 2 kg) were included in the study. After intramuscular premedication with ketamine (20 mg/kg), piritramide (1 mg/kg) and atropine (0.5 mg), anaesthesia was induced with intravenous sodium pentobarbital (12 mg/kg). After endotracheal intubation, anaesthesia was maintained with a continuous intravenous infusion of sodium pentobarbital (3 to 4 mg/kg per hour), sufentanil (3 μg/kg per hour) and pancuronium (0.2 mg/kg per hour). Mechanical ventilation with a mixture of oxygen and room air was adjusted to achieve normocapnia and normoxia, as controlled with arterial blood gas measurements taken at regular intervals (ABL 520; Radiometer A/S, Brønshøj, Denmark). A balanced electrolyte solution was administered at a rate of 8 ml/kg per hour. Normothermia was maintained during the entire procedure using an infrared heating lamp.

A 7.5-Fr central venous catheter was inserted into the femoral vein. A 16-G arterial catheter was advanced into the descending aorta via the femoral artery. A lateral cut-down was performed in the cervical region and an 8.5-Fr introducer sheath was inserted into the left carotid artery.

Via a midline sternotomy, a tourniquet was placed around the inferior vena cava (IVC) for controlled reductions in ventricular preload. A 20 mm nonrestricting perivascular flow probe (Transonic Systems Inc., Ithaca, NY, USA) was placed around the main pulmonary artery (PA). A 6-Fr micro-tipped pressure transducer (SPC 360; Millar Instruments, Houston, TX, USA) was advanced into the PA via a stab wound in the pulmonary outflow tract with its tip just distal to the flow probe. Combined micro-tip multisegment pressure-volume catheters (SPC 560 and SPC 570; Millar Instruments) were inserted into the right ventricle and left ventricle, through a stab wound in the pulmonary outflow tract and via the left carotid artery, respectively. Correct position of the conductance catheters was confirmed with radiography.

### Experimental protocol

Haemodynamic measurements were started after completion of instrumentation and 30 minutes of haemodynamic stabilization. Measurements were always performed with the ventilation suspended at end-expiration. Data were acquired during steady-state conditions and during a brief period of IVC occlusion to obtain a series of successive heart beats at progressively lower end-diastolic volumes, for the calculation of contractile indices and pulmonary pressure-flow relationships.

In 16 animals acute PHT was induced with hypoxic pulmonary vasoconstriction. After control haemodynamic measurements, nitrogen was added to the inspiratory gas mixture and the fraction of inspired oxygen reduced until the mean PA pressures exhibited an increase of at least 50% compared with baseline values (for a detailed description of the ventilator settings and the arterial blood gas status, see Table 1). When stable haemodynamic conditions were achieved in hypoxia, haemodynamic measurements were repeated. Pigs were then randomly assigned to two groups: one group (n = 8) received inhaled iloprost (50 μg dissolved in 5 ml isotonic saline solution), whereas the other group (n = 8) underwent inhalation of placebo (5 ml isotonic saline solution).

**Table 1:** Respirator settings and arterial blood gas status in animals subjected to acute pulmonary hypertension

Parameter	Treatment	Baseline	Pulmonary hypertension	
			Pre-inhalation	5 minutes after inhalation
RR (breaths/minute)	Iloprost	17 ± 1	17 ± 1	17 ± 1
	Control	17 ± 1	17 ± 1	17 ± 2
V_T _(ml/kg)	Iloprost	10 ± 1	10 ± 1	10 ± 1
	Control	11 ± 1	10 ± 1	10 ± 1
FiO_2 _(%)	Iloprost	37 ± 9	15 ± 2*	15 ± 2*
	Control	35 ± 8	15 ± 1*	15 ± 1*
PO_2 _(mmHg)	Iloprost	161 ± 38	43 ± 4*	43 ± 4*
	Control	161 ± 46	47 ± 5*	44 ± 5*
PCO_2 _(mmHg)	Iloprost	38 ± 2	38 ± 2	41 ± 4
	Control	40 ± 4	37 ± 2	39 ± 3
PH	Iloprost	7.46 ± 0.06	7.45 ± 0.07	7.41 ± 0.08
	Control	7.45 ± 0.04	7.47 ± 0.06	7.47 ± 0.04

Values are shown for baseline and in pulmonary hypertension before inhalation and 5 minutes after inhalation of either iloprost or control. For the complete experimental time course, see Additional file 1. Values are expressed as mean ± standard deviation. *P < 0.05 versus baseline (corrected for multiple comparisons). FiO_2_, fraction of inspired oxygen; P(C)O_2_, arterial partial pressure of oxygen (carbon dioxide); RR, respiratory rate; V_T_, tidal volume.

In a subsequent study, including 10 pigs, the effects of inhaled iloprost (Ilomedin^®^; Schering Deutschland GmbH, Berlin, Germany; 50 μg dissolved in 5 ml isotonic saline solution) were examined with (n = 5) and without (n = 5) blockade of the ANS. Blockade of the ANS was accomplished with atropine methyl nitrate (3 mg/kg), propranolol hydrochloride (2 mg/kg) and hexamethonium bromide (20 mg/kg; Sigma-Aldrich NV/SA, Bornem, Belgium).

Haemodynamic and blood gas measurements were performed at the following time points: baseline, and 1, 5, 10 and 30 minutes after stopping iloprost/placebo inhalation. In the PHT group, measurements at 1 minute after stopping iloprost/placebo inhalation consisted only of the registration of steady-state haemodynamics, in order not to destabilize the animals with too frequent IVC occlusions during PHT.

At the end of the experiments, the animals were killed with intravenously administered potassium chloride during deep anaesthesia.

Both iloprost and the placebo solution were aerosolized using a commercially available ultrasonic nebulizer (OPTINEB^®^-ir; NEBU-TEC med. Produkte Eike Kern GmbH, Elsenfeld, Germany) connected to the inspiratory limb of the ventilator circuit. This nebulizer is characterized by a mass median aerodynamic diameter of 2.3 μm and a geometric standard deviation of 1.6. Nebulization times were not prefixed as in the chosen mode of delivery; the device stops automatically when aerosolization of the volume filled into the nebulizer is completed. Mean nebulization times in our experimental setting were 617 ± 67 seconds. After stopping iloprost/placebo inhalation, an average fluid amount of 0.50 ± 0.12 ml was found to remain in the nebulizer.

### Data acquisition

Each conductance catheter was connected to a signal-processing unit (Sigma 5 DF; CDLeycom, Zoetermeer, The Netherlands), in one of which the excitation frequency had been adjusted from 20 to 19 kHz in order to avoid cross-talk [18]. The theory of conductance volumetry has previously been described extensively [19]. Parallel conductance was measured by injecting 10 ml hypertonic saline into the right atrium [20] and blood resistivity was determined. The correction factor α was re-calculated for each measurement.

All parameters were digitized at 333 Hz and stored for off-line analysis with custom-made algorithms written in Matlab^® ^(The Mathworks Inc., Natick, MA, USA).

### Data analysis

**Figure 1:**
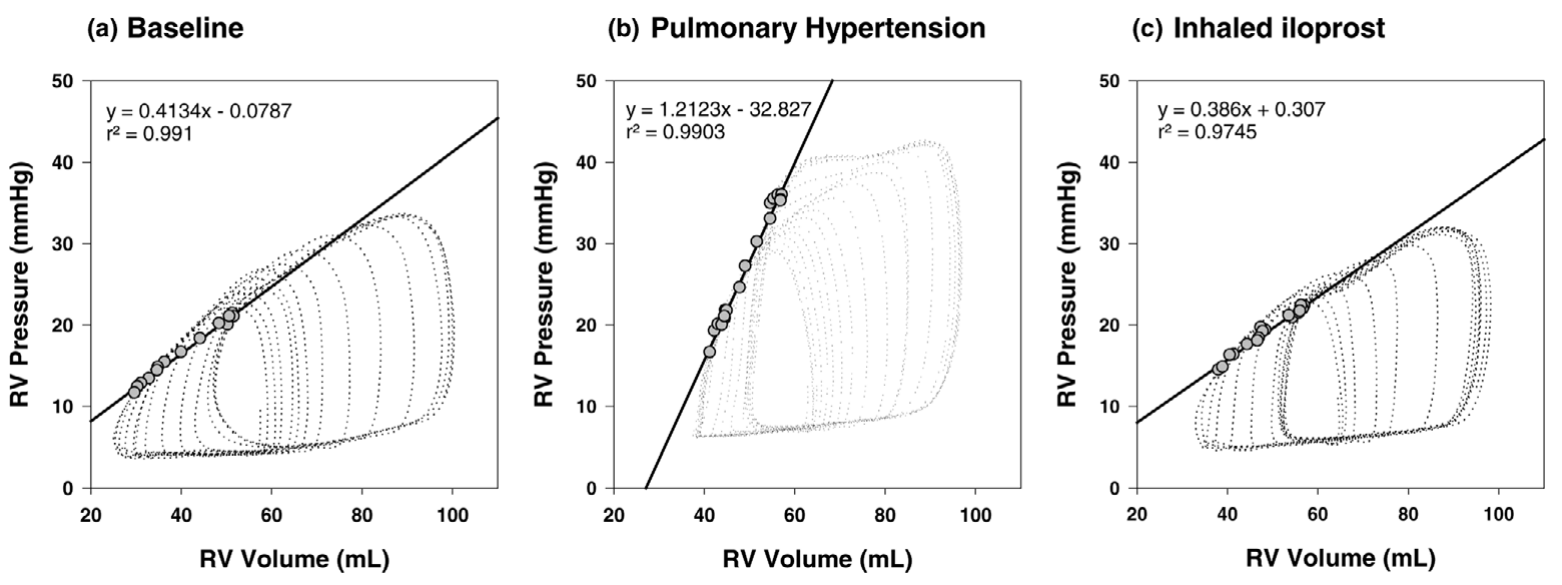
Assessment of right ventricular contractility by pressure-volume loop analysis. Presented are RV pressure-volume loops (dotted lines) in one representative animal at **(a) **baseline, in **(b) **pulmonary hypertension, and **(c) **5 minutes after inhalation of iloprost. These were obtained during a controlled preload reduction by occlusion of the inferior caval vein. The end-systolic pressure-volume relationship is obtained by fitting a regression line (solid line) through the points of maximal (end-systolic) elastance, delineated for each cardiac cycle with grey circles. The induction of pulmonary hypertension elicits an immediate increase in RV contractility (as indicated by the increase in the slope of the end-systolic pressure-volume relationship), which serves the right ventricle to preserve pump performance without changing preload in the face of high afterload conditions (homeometric autoregulation). Conversely, treatment of pulmonary hypertension with inhaled iloprost obviates the need for homeometric autoregulation and allows the right ventricle to return to its baseline (lower) contractile state. RV, right ventricular.

As previously described, ventricular contractility was quantified with the slope of the end-systolic pressure-volume relationship (Emax; Figure 1) and the slope of the preload-recruitable stroke work relationship (Mw) [21,22]. Myocardial energetics of the right ventricle were assessed with computation of the pressure-volume area (PVA). PVA was calculated as the sum of stroke (external) work (area within the pressure-volume loop) and potential energy (area under the end-systolic pressure-volume line on the origin side of the pressure-volume loop; see Additional file 2) [23]. Diastolic function was analyzed using the heart-rate corrected time constant of isovolumetric ventricular relaxation τ [24] and the chamber stiffness constant β [25]. Right coronary artery perfusion pressure was estimated as the difference between systolic arterial pressure and RV systolic pressure [26]. As an estimate for RV oxygen demand, we calculated the rate-pressure product (RPP). Both PVA [27] and RPP have been demonstrated to exhibit excellent correlation with measured oxygen consumption in the right ventricle [28].

Ventricular afterload was quantified as effective arterial elastance (Ea; the ratio of end-systolic pressure to stroke volume). Ventriculo-vascular coupling was described as the ratio of Emax over Ea [29]. PA compliance was calculated using the pulse pressure method [30]. PA resistance was determined using pressure-flow (PQ) plots that allow discrimination between passive (flow induced) and active (tone induced) changes in PA pressures [31]. Pulmonary PQ relations were obtained by plotting, for every heart beat, the mean PA pressure over cardiac output during the rapid flow reduction induced by IVC occlusion. The slopes of the resulting PQ plots were analyzed by linear regression [31]. Vascular resistances were calculated as pressure gradients over mean flow. PA impedance was determined from Fourier series expressions of pressure and flow.

### Statistical analysis

The sample size was calculated based on previous experiments. Power analysis revealed a minimal sample size of five pigs to detect a 33% effect in mean PA pressure and RV-Mw, when a level of significance of 0.05 and a power of 80% were to be achieved. Results were statistically analyzed using a commercially available software package (Statistica^© ^for Windows, version 6.0; Statsoft, Tulsa, OK, USA). To test the global hypothesis that iloprost has an effect on haemodynamic variables in PHT, the group versus time interaction was analyzed using repeated measures analysis of variance with the within-factor time and the grouping factor treatment (iloprost versus control). Likewise, the effects of ANS blockade were tested with the grouping factor autonomic blockade versus no ANS blockade [32]. In case of significant results, horizontal and vertical pair-wise contrasts were performed using the paired and unpaired Student's *t*-test, respectively. The Bonferroni-Holm adjustment was used to correct for multiple comparisons [33]. Nonparametric data were analyzed using Friedman's analysis of variance, the Mann-Whitney U-test and the Wilcoxon signed rank test.

In all conditions, a *P *value < 0.05 was considered statistically significant.

## Results

### Haemodynamic effects of hypoxia-induced pulmonary hypertension

**Figure 2:**
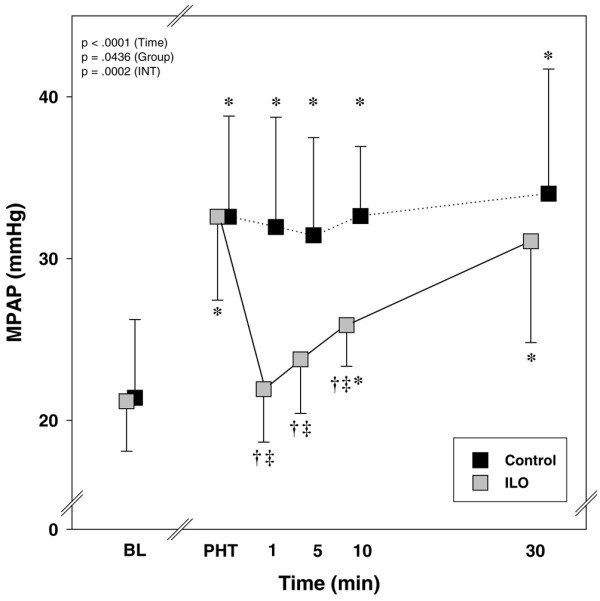
The effects of inhaled iloprost on MPAP. The panels show the characteristic experimental time course, with a maximum pulmonary vasodilating effect immediately after inhalation and a duration of action of approximately 30 minutes. The data are expressed as mean ± standard deviation. *P < 0.05 versus BL; ^†^*P *< 0.05 versus before inhalation; ^‡^*P *< 0.05, ILO versus C (adjusted for multiple comparisons). In addition, *P *values of the repeated measures analysis of variance are shown separately for the time, group and interaction (time × group) effects. BL, baseline; C, control; ILO, iloprost; INT, interaction; MPAP, mean pulmonary artery pressure; PHT, pulmonary hypertension.

Alveolar hypoxia caused an increase in mean PA pressure (Figure 2), calculated pulmonary vascular resistance (PVR) and heart rate, whereas mean arterial pressure and systemic vascular resistance decreased. The ratio of PVR to systemic vascular resistance exhibited a nearly threefold increase from baseline conditions (Table 2).

**Table 2:** General haemodynamics in animals subjected to acute pulmonary hypertension

Parameter	Treatment	Baseline	Pulmonary hypertension	
			Pre-inhalation	5 minutes after inhalation
HR (beats/minute)	Iloprost	88 ± 12	112 ± 13*	109 ± 7*
	Control	90 ± 16	111 ± 14*	116 ± 15*
CO (l/minute)	Iloprost	4.5 ± 0.5	5.0 ± 0.4	5.6 ± 0.9^‡^
	Control	4.1 ± 0.9	4.3 ± 0.6	3.9 ± 1.0
SV (ml)	Iloprost	53 ± 6	45 ± 6	51 ± 9^‡^
	Control	45 ± 13	40 ± 10	35 ± 11
MAP (mmHg)	Iloprost	86 ± 18	69 ± 16*	74 ± 17*
	Control	81 ± 7	70 ± 7*	70 ± 11*
LVEDP (mmHg)	Iloprost	11 ± 2	10 ± 2	11 ± 2
	Control	11 ± 3	10 ± 3	11 ± 2
RVEDP (mmHg)	Iloprost	10 ± 1	11 ± 1	10 ± 2
	Control	10 ± 2	12 ± 1	12 ± 1
SVR (dyn·s/cm^5^)	Iloprost	1,329 ± 324	952 ± 337*	971 ± 406*
	Control	1,518 ± 344	1,087 ± 224*	1,257 ± 368*
PVR (dyn·s/cm^5^)	Iloprost	178 ± 79	366 ± 126*	193 ± 66^†‡^
	Control	210 ± 105	448 ± 168*	464 ± 201*
PVR/SVR	Iloprost	0.14 ± 0.06	0.39 ± 0.07*	0.21 ± 0.09^†^
	Control	0.13 ± 0.05	0.41 ± 0.12*	0.37 ± 0.12*

Values are shown for baseline and in pulmonary hypertension before inhalation and 5 minutes after inhalation of either iloprost or control. For the complete experimental time course, see Additional file 3. Values are expressed as mean ± standard deviation. *P < 0.05 versus baseline; ^†^*P *< 0.05 versus before inhalation; ^‡^*P *< 0.05 iloprost versus control (corrected for multiple comparisons). CO, cardiac output; HR, heart rate; L(R)VEDP, left (right) ventricular end-diastolic pressure; MAP, mean arterial pressure; S(P)VR, systemic (pulmonary) vascular resistance; SV, stroke volume.

**Figure 3:**
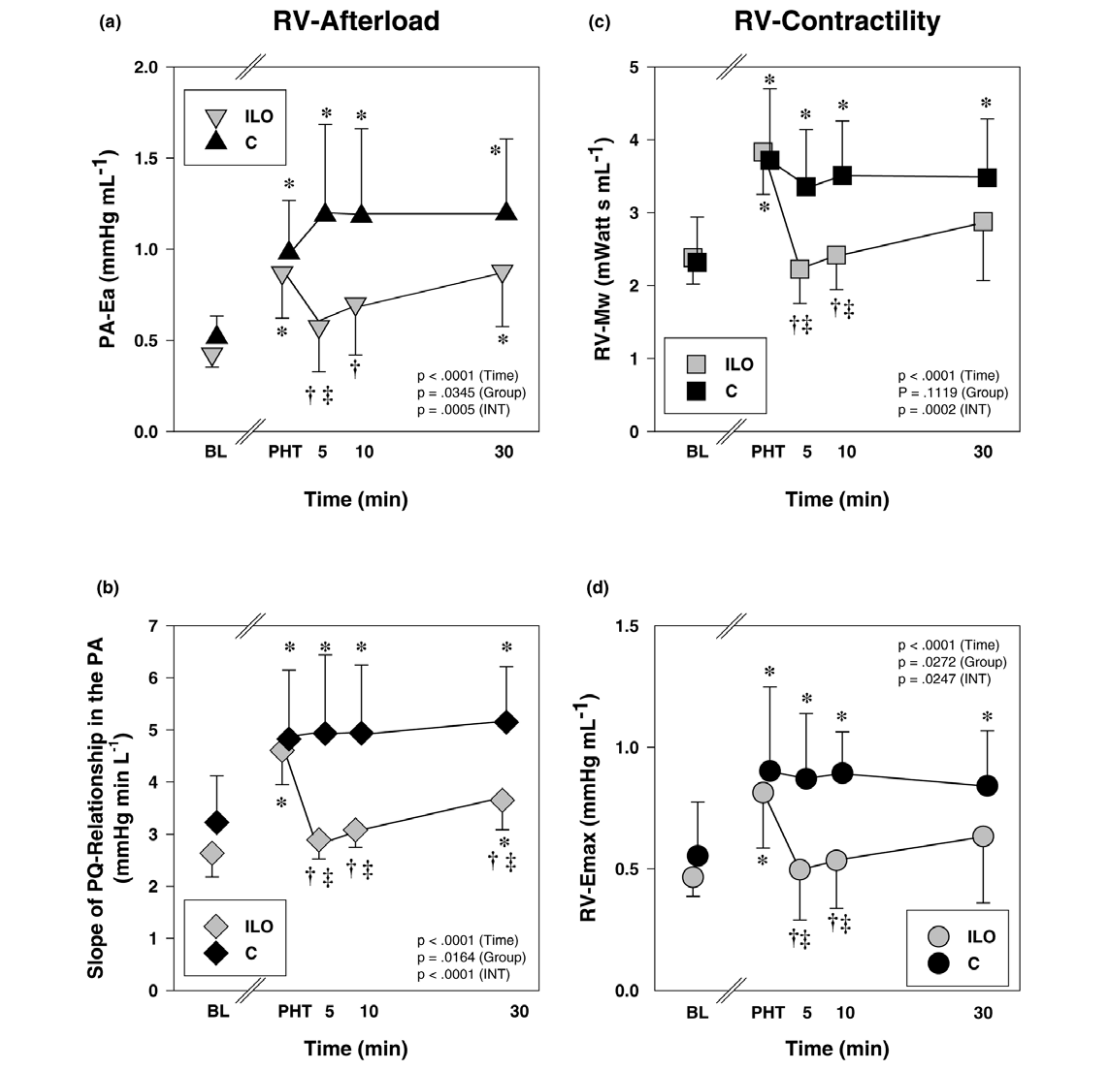
The effects of inhaled iloprost on RV afterload and contractility in animals with PHT. **(a) **RV afterload is illustrated with effective pulmonary arterial elastance (PA-Ea), and **(b) **the slopes of the PQ relationships in the PA. RV contractility is shown as **(c) **Mw and **(d) **Emax. Data are expressed as mean ± standard deviation. *P < 0.05 versus BL; ^†^*P *< 0.05 versus before inhalation; ^‡^*P *< 0.05, ILO versus C (adjusted for multiple comparisons). In addition, *P *values of the repeated measures analysis of variance are shown separately for the time, group and INT (time × group) effects. BL, baseline; C, control; Ea, effective arterial elastance; Emax, slope of the end-systolic pressure-volume relationship; ILO, iloprost; INT, interaction; Mw, slope of the preload-recruitable stroke work relationship; PHT, pulmonary hypertension; PA, pulmonary artery; PQ, pressure-flow; RV, right ventricular.

**Figure 4:**
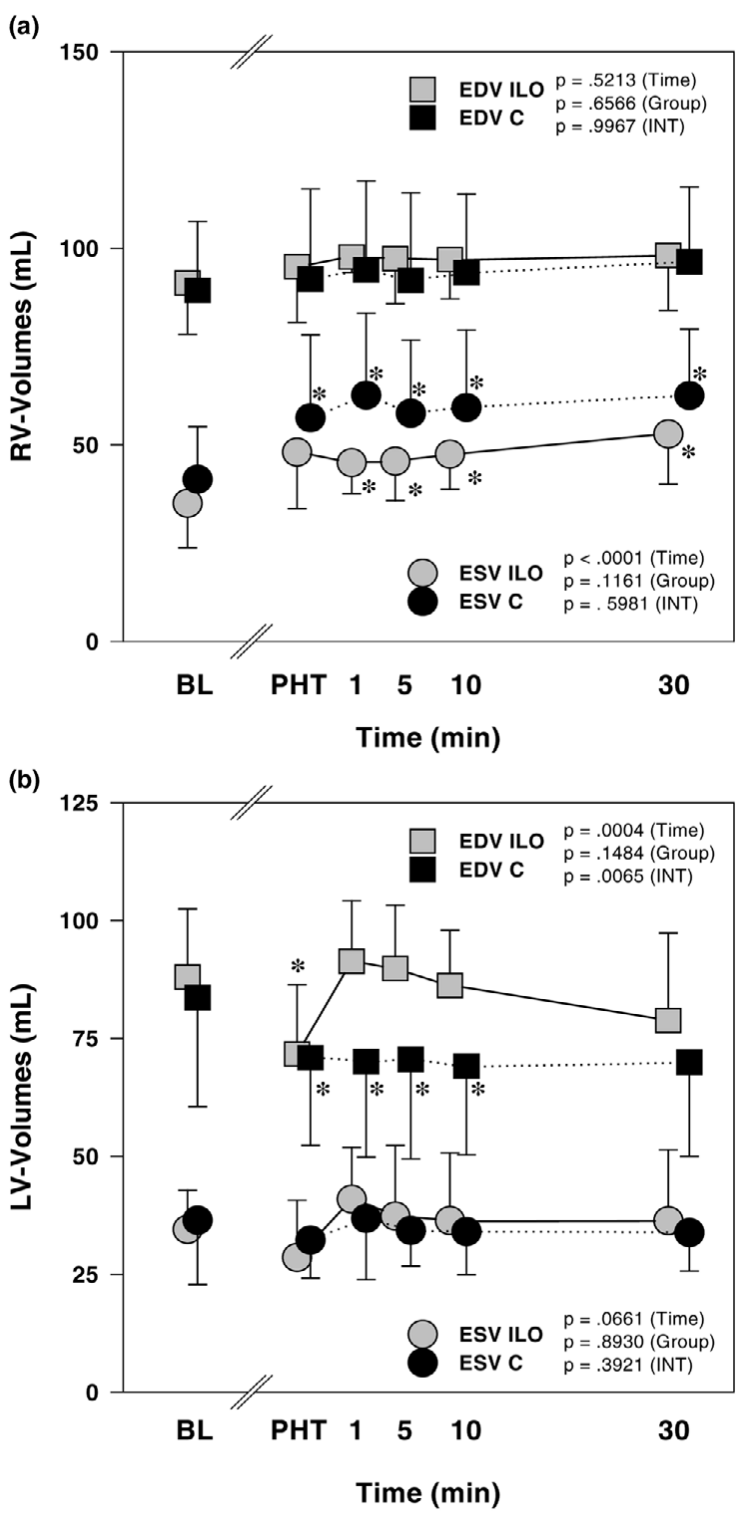
The effects of inhaled iloprost on ventricular interdependence. Shown are the effects of inhaled iloprost on end-diastolic and endsystolic volumes in the **(a) **right ventricle and **(b) **left ventricle in animals with PHT. Data are expressed as mean ± standard deviation. *P < 0.05 versus BL; ^†^*P *< 0.05 versus before inhalation; ^‡^*P *< 0.05, ILO versus C (adjusted for multiple comparisons). In addition, *P *values of the repeated measures analysis of variance are shown separately for the time, group and INT (time × group) effects. BL, baseline; EDV, end-diastolic volume; ESV, end-systolic volume; ILO, iloprost; INT, interaction; LV, left ventricular; PHT, pulmonary hypertension; RV, right ventricular.

An increase in RV afterload was indicated by a higher effective PA-Ea (Figure 3a), a lower PA compliance (Table 3) and a steeper slope of the PA PQ relationship (Figure 3b). RV contractility was also higher in the presence of PHT, because both Mw (Figure 3c) and Emax (Figure 3d) increased from baseline conditions. Hence, ventriculo-vascular coupling (defined as the quotient of Emax over PA-Ea) was preserved at essentially the same level as in healthy animals (Table 3). However, there was also a reduction in RV coronary artery perfusion pressure in the presence of increased oxygen consumption, indicated by the elevated RV RPP and PVA (Table 4). RV ejection fraction decreased (Table 3) as a result of an increase in RV end-systolic volumes and unchanged RV end-diastolic volumes (Figure 4a). RV diastolic function was not affected by induction of PHT, as indicated by isovolumic relaxation and chamber stiffness (Table 3).

**Table 3:** Conductance catheter derived parameters of right ventricular function in animals subjected to acute pulmonary hypertension

Parameter	Treatment	Baseline	Pulmonary hypertension	
			Pre-inhalation	5 minutes after inhalation
REF (%)	Iloprost	62 ± 7	50 ± 8*	53 ± 8* ^‡^
	Control	54 ± 10	40 ± 11*	37 ± 10*
τ/RR	Iloprost	0.08 ± 0.01	0.08 ± 0.01	0.09 ± 0.01^†^
	Control	0.07 ± 0.01	0.07 ± 0.01	0.07 ± 0.01
β (ml^-1^)	Iloprost	0.02 ± 0.01	0.02 ± 0.01	0.02 ± 0.01
	Control	0.02 ± 0.02	0.02 ± 0.01	0.02 ± 0.01
C (ml/mmHg)	Iloprost	2.29 ± 0.49	1.28 ± 0.33*	1.89 ± 0.64^†^
	Control	2.11 ± 0.93	1.08 ± 0.33*	1.11 ± 0.33*
Emax/Ea	Iloprost	1.12 ± 0.11	1.29 ± 0.29	1.03 ± 0.15
	Control	1.11 ± 0.46	1.01 ± 0.31	0.97 ± 0.33

Values are shown for baseline and in pulmonary hypertension before inhalation and 5 minutes after inhalation of either iloprost or control. For the complete experimental time course, see Additional file 4. Values are expressed as mean ± standard deviation. *P < 0.05 versus baseline; ^†^*P *< 0.05 versus before inhalation; ^‡^*P *< 0.05 iloprost versus control (corrected for multiple comparisons). β = chamber stiffness constant of end-diastolic pressure volume relationship; C, pulmonary artery compliance; Emax/Ea, ratio of the slope of the end-systolic pressure-volume relationship to effective pulmonary arterial elastance; REF, right ventricular ejection fraction; τ/RR, time constant of ventricular relaxation, corrected for the RR interval.

**Table 4:** Parameters of right ventricular oxygen balance in animals subjected to acute pulmonary hypertension

Parameter	Treatment	Baseline	Pulmonary hypertension	
			Pre-inhalation	5 minutes after inhalation
RCA-PP (mmHg)	Iloprost	86 ± 15	61 ± 22*	78 ± 18* ^†‡^
	Control	73 ± 13	51 ± 9*	53 ± 12*
RPP (bpm·mmHg)	Iloprost	2,105 ± 342	4,390 ± 766*	2,904 ± 727* ^†‡^
	Control	2,343 ± 816	4,373 ± 997*	4,414 ± 995*
ΔHR·PVA (%)	Iloprost	100 ± 0	191 ± 66*	122 ± 29^†‡^
	Control	100 ± 0	188 ± 44*	197 ± 49*

Values are shown for baseline and in pulmonary hypertension before inhalation and 5 minutes after inhalation of either iloprost or control. For the complete experimental time course, see Additional file 5. Values are expressed as mean ± standard deviation. *P < 0.05 versus baseline; ^†^*P *< 0.05 versus before inhalation; ^‡^*P *< 0.05 iloprost versus control (corrected for multiple comparisons). bpm, beats/minute; ΔHR·PVA, changes in the product of heart rate and pressure-volume area; RCA-PP, right coronary artery perfusion pressure; RPP, right ventricular rate pressure product.

In the left ventricle, Mw increased during hypoxia-induced PHT whereas Ea was no different from baseline (Table 5). Finally, PHT caused a significant reduction in left ventricular (LV) end-diastolic volumes (Figure 4b) whereas parameters of LV diastolic function were not significantly different from control (Table 5).

**Table 5:** Conductance catheter derived parameters of left ventricular function in animals subjected to acute pulmonary hypertension

Parameter	Treatment	Baseline	Pulmonary hypertension	
			Pre-inhalation	5 minutes after inhalation
Mw (mWatt·s/ml)	Iloprost	8.29 ± 1.66	10.67 ± 2.45*	8.08 ± 2.22
	Control	7.54 ± 1.06	9.01 ± 1.27*	9.24 ± 2.69
Emax (mmHg/ml)	Iloprost	1.10 ± 0.46	1.51 ± 0.74	1.20 ± 0.66
	Control	1.13 ± 0.62	1.29 ± 0.75	1.64 ± 0.95
LVEF (%)	Iloprost	61 ± 5	63 ± 12	59 ± 13
	Control	57 ± 9	53 ± 7	50 ± 5
τ/RR (ms)	Iloprost	0.06 ± 0.02	0.08 ± 0.02	0.07 ± 0.01
	Control	0.07 ± 0.01	0.07 ± 0.01	0.08 ± 0.02
β (ml^-1^)	Iloprost	0.11 ± 0.03	0.11 ± 0.06	0.04 ± 0.03*
	Control	0.11 ± 0.04	0.13 ± 0.09	0.09 ± 0.05
C (ml/mmHg)	Iloprost	0.72 ± 0.23	0.60 ± 0.20	0.64 ± 0.27
	Control	0.88 ± 0.27	0.71 ± 0.25*	0.66 ± 0.21*
Ea (mmHg/ml)	Iloprost	1.87 ± 0.51	1.68 ± 0.44	1.67 ± 0.50
	Control	1.80 ± 0.46	1.93 ± 0.55	1.95 ± 0.56
Emax/Ea	Iloprost	0.60 ± 0.24	0.92 ± 0.50	0.69 ± 0.30
	Control	0.62 ± 0.24	0.67 ± 0.31	0.86 ± 0.48

Values are shown for baseline and in pulmonary hypertension before inhalation and 5 minutes after inhalation of either iloprost or control. For the complete experimental time course, see Additional file 6. Values are expressed as mean ± standard deviation. *P < 0.05 versus baseline. β, chamber stiffness constant of end-diastolic pressure volume relationship; C, aortic compliance; Ea, effective arterial elastance; Emax, slope of the end-systolic pressure-volume relationship; LVEF, left ventricular ejection fraction; Mw, slope of the preload-recruitable stroke work relationship; τ/RR, time constant of ventricular relaxation, corrected for the RR interval.

### Haemodynamic effects of inhaled iloprost in hypoxia-induced pulmonary hypertension

Inhalation of iloprost rapidly restored mean PA pressure and PVR to baseline values (Figure 2 and Table 2). Animals treated with iloprost had a significantly higher cardiac output and mean arterial pressure than did animals in the control group.

RV afterload was significantly decreased, as indicated by a lower PA-Ea, a higher PA compliance (Figure 3a and Table 3) and significant reduction in the slopes of the PQ relationships (Figure 3b). This was accompanied by a reduction in RV contractility; both Mw and Emax decreased as compared with the untreated PHT condition (Figure 3c,d), which resulted in a preservation of ventriculo-vascular coupling (Emax/Ea; Table 3). However, in iloprost-treated animals, oxygen supply-demand balance was now significantly improved; right coronary artery perfusion pressure was higher and RV RPP and RV PVA were lower as compared with untreated PHT animals (Table 4). RV ejection fraction was significantly improved by inhaled iloprost, whereas RV end-diastolic volumes and diastolic function were not affected (Figure 4a and Table 3).

LV contractility, diastolic function and afterload were not affected (Table 5), but LV end-diastolic volumes were significantly increased after iloprost (Figure 4b).

### Haemodynamic effects of inhaled iloprost in healthy animals

**Figure 5:**
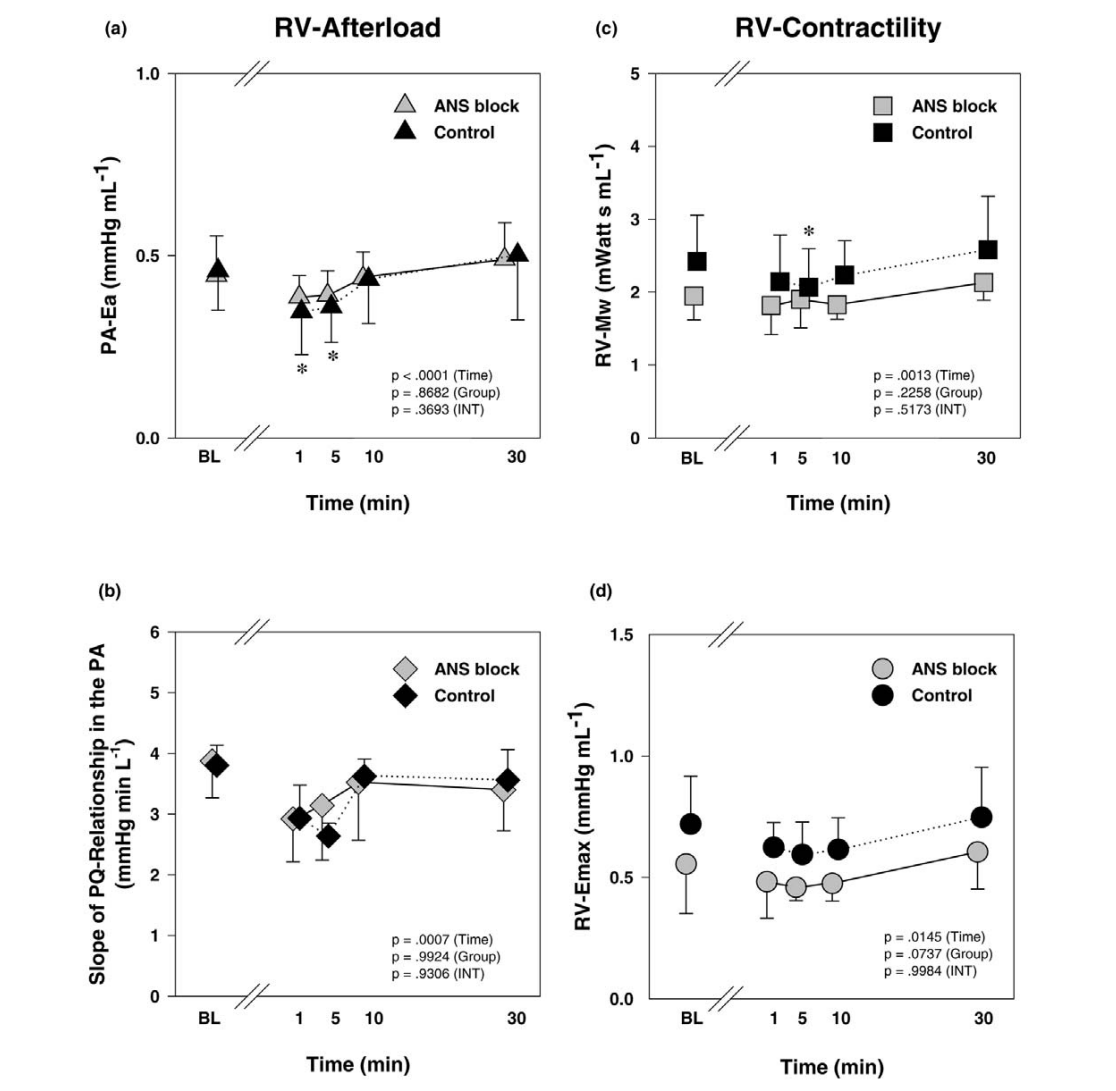
The effects of inhaled iloprost on RV afterload and contractility in animals with and without blockade of the ANS. RV afterload is illustrated by **(a) **effective pulmonary arterial elastance (PA-Ea) and **(b) **the slopes of the PQ relationships in the PA. RV contractility is shown as **(c) **Mw and **(d) **Emax. Data are expressed as mean ± standard deviation. *P < 0.05 versus BL (adjusted for multiple comparisons). In addition, *P *values of the repeated measures analysis of variance are shown separately for the time, group and INT (time × group) effects. ANS, autonomous nervous system; BL, baseline; Ea, effective arterial elastance; Emax, slope of the end-systolic pressure-volume relationship; INT, intraction; Mw, slope of the preload-recruitable stroke work relationship; PA, pulmonary artery; PQ, pressure-flow; RV, right ventricular.

In undiseased conditions, inhalation of iloprost resulted in a mild but statistically significant decrease in RV afterload, whereas other haemodynamic parameters exhibited no major changes (Figure 5a,b; for a detailed description of haemodynamics in this subset of animals, see Additional file 7). This was associated with a small reduction in contractility, indicated by both RV Mw and Emax (Figure 5c,d). These effects were comparable in animals with and without ANS blockade. Parameters of LV afterload and contractility remained unchanged after the inhalation of iloprost (data not shown).

## Discussion

Our data confirm that inhaled iloprost improves cardiovascular performance in the presence of acute PHT, primarily through a selective reduction in RV afterload. Interestingly, the pulmonary vasodilator effects of inhaled iloprost were invariably associated with a reduction in RV contractility. This is directionally opposite to what *in vitro *experiments have suggested earlier, namely that prostacylins may possess positive inotropic properties by stimulating adenyl cyclase activity in cardiomyocytes [34,35].

*In vivo *work in this area has never provided convincing evidence for a direct positive inotropic effect of PGI_2_. In clinical studies, such claims were based on load-dependent haemodynamic indices [6,36]. In experimental studies conducted in pigs, supraclinical doses of intravenous iloprost caused an elevated contractile state but also systemic hypotension and tachycardia [7], so that the rise in contractility possibly resulted from baroreflex-mediated sympathetic activation [37]. Another group found no measurable effect of intravenous PGI_2 _on contractility using a canine model of load-induced RV dysfunction [9]. We, in contrast, recently reported a dose-dependent decrease in RV contractility after intravenous administration of epoprostenol in pigs with acute PHT [8]. We hypothesized that this reduced inotropic state was related to tight coupling between RV afterload and contractility. Indeed, in a variety of animal models, but also in humans, it was shown that acute and chronic PHT elicit an immediate increase in RV contractility [38].

This reflex mechanism is referred to as 'homeometric autoregulation' and is postulated to result from stimulation of stretch-activated calcium channels [39], release of positive inotropic substances from the endocardial endothelium [40] and/or elevated sympathetic tone [21,41]. Homeometric autoregulation serves the right ventricle to preserve pump performance without changing preload in the face of high afterload conditions. Conversely, alleviation of PHT with any effective pulmonary vasodilator obviates the need for homeometric autoregulation and allows the right ventricle to return to its baseline (lower) contractile state. This could be mistaken for a drug-induced negative inotropic effect. A similar phenomenon has been described after treatment of PHT with inhaled NO [42]. The discrepancy between our findings and those reported previously by Kerbaul and coworkers [9], who did not observe an increase in contractility with PHT or a decrease after intravenous PGI_2 _treatment, is probably related to differences in the experimental model; dogs had depressed RV function before treatment in that study.

Hence, the negative inotropic effects observed during iloprost inhalation in PHT are similar to our previous findings with intravenous PGI_2 _[8]. This observation favours the hypothesis that the reduction in contractility is an indirect phenomenon caused by the immediate adaptation of RV contractility to match a drug-induced reduction in RV afterload. However, basing this hypothesis solely on findings during PHT may be delusive, because we could not entirely rule out the possibility that inhaled iloprost might exert a subtle positive inotropic action, which could have been masked or counteracted by the predominant effects on RV afterload. We therefore repeated the experiments in undiseased animals in which pharmacologically induced pulmonary vasodilatation was less pronounced. Still, we found RV contractility to parallel RV afterload closely (and not to be increased by iloprost). Interestingly, this mechanism occurred even in ANS blocked animals, suggesting that the well known interaction of prostanoids with the ANS did not contribute to our observations.

It appears from our data that matching contractility to the prevailing afterload allowed the right ventricle to preserve global pump performance at lower energetic cost. PVA and RV RPP, both estimates of RV oxygen consumption [23,28], normalized almost to baseline levels after iloprost treatment in animals with PHT. Right coronary artery perfusion pressure increased, indicating a simultaneous improvement in RV oxygen supply. It is tempting to speculate that such an energy-conserving mechanism contributes to the beneficial effects of iloprost in the treatment of patients with chronic PHT [1].

The improvement in global haemodynamics by inhaled iloprost can also, at least partly, be attributed to the phenomenon of ventricular interdependence. The latter is known to play a key role in the disruption of circulatory homeostasis during PHT. The pressure overloaded right ventricle eventually distends and has a direct impact on LV performance through serial (failure to produce antegrade filling of the left ventricle) and parallel (disturbance of diastolic and systolic LV function by leftward shifting of the septum) ventricular interaction [43,44]. In fact, reducing RV pressure load with iloprost allowed the immediate restoration of LV filling after inhalation of iloprost.

### Limitations of the study

Several limitations of the present study should be acknowledged.

Data were obtained in an open chest-open pericardium model using general anaesthesia. These experimental conditions may significantly affect cardiovascular mechanics, but we considered them relevant to the setting of cardiac surgery, in which RV dysfunction is an important risk factor for perioperative mortality [45]. In addition, opening of the pericardium does not interfere with serial ventricular interaction. In PHT, series interaction has been demonstrated to account for 65% of the decrease in LV preload, even after relief of pericardial constraint [46]. Acute PHT was created by inducing hypoxic pulmonary vasoconstriction, and this may not be representative for clinical cases of PHT that are unrelated to hypoxia. It is particularly important to note that in our study healthy pigs exhibited an ability to increase contractile performance when they were subjected to hypoxia, whereas in clinical practice contractile performance and/or contractile reserve of the right ventricle is often impaired. However, recently published data obtained in a model of RV failure appear to be in accordance with our observation, namely that prostacyclins are devoid of direct positive inotropic effects [9]. The optimal dosage for iloprost therapy in acute PHT remains unknown, because no dose-response curves are available for this particular situation, but the selected dose and time in our study is within the range reported in the literature for humans [15] and pigs [47].

RV coronary perfusion pressure was calculated rather than measured directly. Because no consensus exists on this matter, we opted to use the difference between systolic arterial pressure and RV systolic pressure, taking into consideration the fact that an important part of right coronary artery flow occurs during systole and that, in PHT, RV coronary artery flow is impaired proportionally to RV systolic pressures [48].

Finally, the influence of ANS blockade on the effects of iloprost was not studied in PHT. In pilot experiments, however, ANS blockade in hypoxia-induced PHT was associated with lethal cardiovascular collapse, highlighting the importance of the intact sympathetic nervous system, as shown recently in our laboratory [21]. Moreover, it must be noted that the iloprost-induced effects were rather short lived. The duration of action seen in our study is within the range of observations in medical and surgical patients [4,10], but it contrasts with demonstrated sustained benefits of inhaled iloprost in patients with primary PHT [1]. Recent evidence, however, suggests that the long-term effects of PGI_2 _might not be related simply to vasodilatation but also to other mechanisms involving pulmonary vascular remodeling [49]. In any case, further pharmacodynamic and pharmacokinetic studies are warranted to define the optimal dosage and strategies to prolong the duration of action for inhaled iloprost in the perioperative setting.

## Conclusion

In animals with acute PHT, inhalation of iloprost resulted in selective pulmonary vasodilation, which – in contrast to previous findings with systemic application of PGI_2 _[8] – was associated with an improvement in global haemodynamics and a restoration of LV preload. The reduction of RV afterload was associated with a paradoxical decrease in RV contractility. Our data suggest that this reflects an indirect mechanism by which ventriculo-vascular coupling is maintained at the lowest possible energetic cost. We found no evidence for a direct negative inotropic effect of iloprost.

## Key messages

• In acute PHT inhaled iloprost improves general haemodynamics, primarily through selective pulmonary vasodilatation.

• Inhaled iloprost effectively restores LV preload via the mechanism of ventricular interdependence.

• Inhaled iloprost indirectly decreases RV contractility but preserves right ventriculo-vascular coupling at lower oxygen cost.

## Abbreviations

ANS: autonomic nervous system; Ea: effective arterial elastance; Emax: slope of the end-systolic pressure-volume relationship; IVC: inferior vena cava; LV: left ventricular; Mw: slope of the preload-recruitable stroke work relationship; PA: pulmonary artery; PGI_2_: prostaglandin I_2_; PHT: pulmonary hypertension; PQ: pressure-flow; PVA: pressure-volume area; PVR: pulmonary vascular resistance; RPP: rate-pressure product; RV: right ventricular.

## Competing interests

In the past five years, SR and WB have received honoraria for lectures from the manufacturer of iloprost. From 2003 to 2004, the Department of Anaesthesiology of the University of Aachen held a research grant from Schering AG, the former manufacturer of iloprost.

## Authors' contributions

SR conceived of the study and – together with CM – designed the study, carried out the animal experiments, performed the data acquisition and the statistical analysis, and wrote the manuscript. PC wrote the software algorithms for the data analysis, participated in the statistical analysis and helped to draft the manuscript. WB participated in the study design and coordination. PW is responsible for the final study design, participated in the animal experiments, supported the data acquisition and the statistical analysis, and edited the final manuscript. All authors read and approved the final manuscript.

## Supplementary Material

Additional file 1 Additional file 1 is a table listing the complete experimental time course of the respirator settings and arterial blood gas status in animals subjected to acute PHT. Click here for file

Additional file 2 Additional file 2 is a figure illustrating the assessment of RV myocardial energetics by computation of the PVA. Click here for file

Additional file 3 Additional file 3 is a table listing the complete experimental time course of general haemodynamics in animals subjected to acute PHT. Click here for file

Additional file 4 Additional file 4 is a table listing the complete experimental time course of conductance catheter derived parameters of RV function in animals subjected to acute PHT. Click here for file

Additional file 5 Additional file 5 is a table listing the complete experimental time course of RV oxygen balance in animals subjected to acute PHT. Click here for file

Additional file 6 Additional file 6 is a table listing the complete experimental time course of conductance catheter derived parameters of LV function in animals subjected to acute PHT. Click here for file

Additional file 7 Additional file 7 is a table listing general hemodynamics in animals with and without blockade of the autonomous nervous system. Click here for file
