# Supplementary material for: Effects of inhaled iloprost on right ventricular contractility, right ventriculo-vascular coupling and ventricular interdependence: a randomized placebo-controlled trial in an experimental model of acute pulmonary hypertension
Source: Crit Care. 2008 Sep 10;12(5):R113. doi: 10.1186/cc7005 (PMC2592739; doi:10.1186/cc7005)
Supplement: Additional file 1 — is a table listing the complete experimental time course of the respirator settings and arterial blood gas status in animals subjected to acute PHT. [file cc7005-S1.doc]

**Additional data file 1:**

Respirator Setting and Arterial Blood Gas Status in Animals subjected to Acute Pulmonary Hypertension.

(Pre-inhal. = before inhalation of iloprost, n min = n minutes after inhalation of either iloprost (ILO) or control (C))

|  |  | **Baseline** | | | **Pulmonary Hypertension** | | | | | | | | | | | | | | | ***RMANOVA*** | | |
| --- | --- | --- | --- | --- | --- | --- | --- | --- | --- | --- | --- | --- | --- | --- | --- | --- | --- | --- | --- | --- | --- | --- |
|  |  |  |  |  | **Pre-inhal.** | | | **1 min** | | | **5 min** | | | **10 min** | | | **30 min** | | | *Time* | *Group* | *INT* |
| **RR** | **ILO** | 17 | ± | 1 | 17 | ± | 1 | 17 | ± | 1 | 17 | ± | 1 | 17 | ± | 1 | 17 | ± | 1 | *.1415* | *.8011* | *.1415* |
| **(min^-1^)** | **C** | 17 | ± | 1 | 17 | ± | 1 | 18 | ± | 1 | 17 | ± | 2 | 17 | ± | 1 | 18 | ± | 1 |  |  |  |
| **V_T_** | **ILO** | 10 | ± | 1 | 10 | ± | 1 | 10 | ± | 1 | 10 | ± | 1 | 10 | ± | 1 | 10 | ± | 1 | *.2442* | *.9061* | *.3486* |
| **(ml kg^-1^)** | **C** | 11 | ± | 1 | 10 | ± | 1 | 10 | ± | 1 | 10 | ± | 1 | 10 | ± | 1 | 10 | ± | 1 |  |  |  |
| **FiO_2_** | **ILO** | 37 | ± | 9 | 15 | ± | 2 * | 15 | ± | 2 * | 15 | ± | 2 * | 15 | ± | 2 * | 15 | ± | 1 * | ***<.0001*** | *.1774* | *.8722* |
| (%) | **C** | 35 | ± | 8 | 15 | ± | 1 * | 16 | ± | 1 * | 15 | ± | 1 * | 15 | ± | 1 * | 15 | ± | 1 * |  |  |  |
| **pO_2_** | **ILO** | 161 | ± | 38 | 43 | ± | 4 * | 45 | ± | 9 * | 43 | ± | 4 * | 43 | ± | 5 * | 43 | ± | 5 * | ***<.0001*** | *.7894* | *.4853* |
| (mmHg) | **C** | 161 | ± | 46 | 47 | ± | 5 * | 45 | ± | 6 * | 44 | ± | 5 * | 45 | ± | 6 * | 43 | ± | 4 * |  |  |  |
| **pCO_2_** | **ILO** | 38 | ± | 2 | 38 | ± | 2 | 41 | ± | 4 | 41 | ± | 4 | 42 | ± | 5 | 41 | ± | 3 | *.0578* | *.4471* | *.4195* |
| (mmHg) | **C** | 40 | ± | 4 | 37 | ± | 2 | 40 | ± | 4 | 39 | ± | 3 | 40 | ± | 4 | 41 | ± | 2 |  |  |  |
| **pH** | **ILO** | 7.46 | ± | 0.06 | 7.45 | ± | 0.07 | 7.38 | ± | 0.06 | 7.41 | ± | 0.08 | 7.39 | ± | 0.08 | 7.38 | ± | 0.08 * | ***<.0001*** | *.6187* | *.1338* |
|  | **C** | 7.45 | ± | 0.04 | 7.47 | ± | 0.06 | 7.47 | ± | 0.05 | 7.47 | ± | 0.04 | 7.45 | ± | 0.05 | 7.44 | ± | 0.02 |  |  |  |

Pre-inhal. = before inhalation of iloprost, n min = n minutes after inhalation of either iloprost (ILO) or control (C)

RR = respiratory rate; V_T_ = tidal volume; FiO_2_ = fraction of inspired oxygen; Pa(C)O_2_ = arterial partial pressure of oxygen (carbon dioxide)

Mean ± SD; * = P < 0.05 vs. Baseline (corrected for multiple comparisons)

p-values of the RMANOVA are shown separately for the time-, group- and interaction- (INT, time x group) effects
