# Supplementary material for: Effects of inhaled iloprost on right ventricular contractility, right ventriculo-vascular coupling and ventricular interdependence: a randomized placebo-controlled trial in an experimental model of acute pulmonary hypertension
Source: Crit Care. 2008 Sep 10;12(5):R113. doi: 10.1186/cc7005 (PMC2592739; doi:10.1186/cc7005)

**Additional data file 2:**

Assessment of right ventricular myocardial energetics by computation of the pressure-volume area in a representative animal subjected to pulmonary hypertension. The pressure-volume area was calculated as the sum of stroke (external) work (shaded area within the PV-loop) and potential energy (grey-coloured area under the end-systolic pressure voulme relationship (ESPVR) line on the origin side of the PV-loop).

EDPVR = end-diastolic pressure volume relationship.


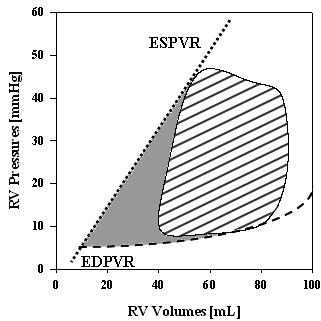

Supplement: Additional file 2 — is a figure illustrating the assessment of RV myocardial energetics by computation of the PVA. [file cc7005-S2.doc]
