# Supplementary material for: Effects of inhaled iloprost on right ventricular contractility, right ventriculo-vascular coupling and ventricular interdependence: a randomized placebo-controlled trial in an experimental model of acute pulmonary hypertension
Source: Crit Care. 2008 Sep 10;12(5):R113. doi: 10.1186/cc7005 (PMC2592739; doi:10.1186/cc7005)
Supplement: Additional file 3 — is a table listing the complete experimental time course of general haemodynamics in animals subjected to acute PHT. [file cc7005-S3.doc]

**Additional data file 3:**

General Hemodynamics in Animals subjected to Acute Pulmonary Hypertension: Complete Experimental Time Course.

|  |  | **Baseline** | | | **Pulmonary Hypertension** | | | | | | | | | | | | | | | ***RMANOVA*** | | |
| --- | --- | --- | --- | --- | --- | --- | --- | --- | --- | --- | --- | --- | --- | --- | --- | --- | --- | --- | --- | --- | --- | --- |
|  |  |  |  |  | **Pre-inhal.** | | | **1 min** | | | **5 min** | | | **10 min** | | | **30 min** | | | *Time* | *Group* | *INT* |
| **HR** | **ILO** | 88 | ± | 12 | 112 | ± | 13 * | 108 | ± | 7 | 109 | ± | 7 * | 110 | ± | 8 | 116 | ± | 14 * | ***<.0001*** | *.4465* | *.6561* |
| (min^-1^) | **C** | 90 | ± | 16 | 111 | ± | 14 * | 114 | ± | 16 * | 116 | ± | 15 * | 117 | ± | 15 * | 122 | ± | 16 * |  |  |  |
| **CO** | **ILO** | 4.5 | ± | 0.5 | 5.0 | ± | 0.4 | 5.6 | ± | 0.7 ‡ | 5.6 | ± | 0.9 ‡ | 5.4 | ± | 0.8 ‡ | 5.0 | ± | 0.9 | *.0566* | ***.0047*** | ***.0013*** |
| (L min^-1^) | **C** | 4.1 | ± | 0.9 | 4.3 | ± | 0.6 | 3.7 | ± | 0.8 | 3.9 | ± | 1.0 | 4.0 | ± | 1.0 | 4.2 | ± | 1.0 |  |  |  |
| **SV** | **ILO** | 53 | ± | 6 | 45 | ± | 6 | 52 | ± | 6 †‡ | 51 | ± | 9 ‡ | 50 | ± | 8 | 44 | ± | 8 | ***.0011*** | ***.0163*** | ***.0032*** |
| (mL) | **C** | 45 | ± | 13 | 40 | ± | 10 | 34 | ± | 11 † | 35 | ± | 11 | 35 | ± | 11 | 35 | ± | 11 |  |  |  |
| **MAP** | **ILO** | 86 | ± | 18 | 69 | ± | 16 * | 74 | ± | 18 *† | 74 | ± | 17 * | 70 | ± | 12 * | 65 | ± | 14 * | ***<.0001*** | *.7256* | ***.0433*** |
| (mmHg) | **C** | 81 | ± | 7 | 70 | ± | 7 * | 66 | ± | 5 * | 70 | ± | 11 * | 68 | ± | 13 * | 70 | ± | 9 * |  |  |  |
| **MPAP** | **ILO** | 21 | ± | 3 | 33 | ± | 5 * | 22 | ± | 3 †‡ | 24 | ± | 3 †‡ | 26 | ± | 2 *†‡ | 31 | ± | 6 * | ***<.0001*** | ***.0436*** | ***.0002*** |
| (mmHg) | **C** | 21 | ± | 5 | 33 | ± | 6 * | 32 | ± | 6 * | 31 | ± | 6 * | 33 | ± | 4 * | 34 | ± | 7 * |  |  |  |
| **LVEDP** | **ILO** | 11 | ± | 2 | 10 | ± | 2 | 10 | ± | 2 | 11 | ± | 2 | 10 | ± | 2 | 11 | ± | 3 | *.1601* | *.8351* | *.7414* |
| (mmHg) | **C** | 11 | ± | 3 | 10 | ± | 3 | 11 | ± | 2 | 11 | ± | 2 | 10 | ± | 2 | 10 | ± | 2 |  |  |  |
| **RVEDP** | **ILO** | 10 | ± | 1 | 11 | ± | 1 | 10 | ± | 2 | 10 | ± | 2 | 10 | ± | 2 | 10 | ± | 3 | *.0841* | *.1227* | *.2012* |
| (mmHg) | **C** | 10 | ± | 2 | 12 | ± | 1 | 12 | ± | 2 | 12 | ± | 1 | 12 | ± | 1 | 12 | ± | 2 |  |  |  |
| **SVR** | **ILO** | 1329 | ± | 324 | 952 | ± | 337 * | 932 | ± | 329 * | 971 | ± | 406 * | 931 | ± | 311 * | 912 | ± | 340 * | ***<.0001*** | *.1826* | *.4546* |
| (dyn s cm^-5^) | **C** | 1518 | ± | 344 | 1087 | ± | 224 * | 1246 | ± | 340 * | 1257 | ± | 368 * | 1177 | ± | 323 * | 1157 | ± | 340 * |  |  |  |
| **PVR** | **ILO** | 178 | ± | 79 | 366 | ± | 126 * | 166 | ± | 57 †‡ | 193 | ± | 66 †‡ | 241 | ± | 63 †‡ | 336 | ± | 154 | ***<.0001*** | ***.0148*** | ***<.0001*** |
| (dyn s cm^-5^) | **C** | 210 | ± | 105 | 448 | ± | 168 * | 501 | ± | 223 * | 464 | ± | 201 * | 510 | ± | 214 * | 477 | ± | 161 * |  |  |  |
| **PVR/SVR** | **ILO** | 0.14 | ± | 0.06 | 0.39 | ± | 0.07 * | 0.19 | ± | 0.07 †‡ | 0.21 | ± | 0.09 † | 0.27 | ± | 0.07 †* | 0.38 | ± | 0.10 * | ***<.0001*** | *.0558* | ***<.0001*** |
|  | **C** | 0.13 | ± | 0.05 | 0.41 | ± | 0.12 * | 0.40 | ± | 0.13 * | 0.37 | ± | 0.12 * | 0.44 | ± | 0.19 * | 0.42 | ± | 0.12 * |  |  |  |

Pre-inhal. = before inhalation, n min = n minutes after inhalation of either iloprost (ILO) or control (C)

HR = heart rate; CO = cardiac output; SV = stroke volume; M(P)AP = mean (pulmonary) arterial pressure; L(R)VEDP = left (right) ventricular enddiastolic pressure; S(P)VR = systemic (pulmonary) vascular resistance

Mean ± SD; * = P < 0.05 vs. Baseline; † = P < 0.05 vs. before inhalation; ‡ = P < 0.05 Iloprost vs. Control (corrected for multiple comparisons)
