# Supplementary material for: Effects of inhaled iloprost on right ventricular contractility, right ventriculo-vascular coupling and ventricular interdependence: a randomized placebo-controlled trial in an experimental model of acute pulmonary hypertension
Source: Crit Care. 2008 Sep 10;12(5):R113. doi: 10.1186/cc7005 (PMC2592739; doi:10.1186/cc7005)
Supplement: Additional file 4 — is a table listing the complete experimental time course of conductance catheter derived parameters of RV function in animals subjected to acute PHT. [file cc7005-S4.doc]

**Additional data file 4:**

Conductance Catheter derived Parameters of Right Ventricular Function in Animals subjected to Acute Pulmonary Hypertension: Complete Experimental Time Course.

|  |  | **Baseline** | | | **Pulmonary Hypertension** | | | | | | | | | | | | | | | ***RMANOVA*** | | |
| --- | --- | --- | --- | --- | --- | --- | --- | --- | --- | --- | --- | --- | --- | --- | --- | --- | --- | --- | --- | --- | --- | --- |
|  |  |  |  |  | **Pre-inhal.** | | | **1 min** | | | **5 min** | | | **10 min** | | | **30 min** | | | *Time* | *Group* | *INT* |
| **REF** | **ILO** | 62 | ± | 7 | 50 | ± | 8 * | 54 | ± | 7 *‡ | 53 | ± | 8 *‡ | 51 | ± | 7 * | 47 | ± | 8 * | ***<.0001*** | ***.0091*** | ***.0323*** |
| (%) | **C** | 54 | ± | 10 | 40 | ± | 11 * | 35 | ± | 9 *† | 37 | ± | 10 * | 37 | ± | 13 * | 38 | ± | 12 * |  |  |  |
| **τ /RR** | **ILO** | 0.08 | ± | 0.01 | 0.08 | ± | 0.01 | 0.09 | ± | 0.01 *† | 0.09 | ± | 0.01 † | 0.09 | ± | 0.01 | 0.08 | ± | 0.01 | ***.0056*** | *.1527* | *.2136* |
|  | **C** | 0.07 | ± | 0.01 | 0.07 | ± | 0.01 | 0.08 | ± | 0.01 | 0.07 | ± | 0.01 | 0.08 | ± | 0.01 | 0.08 | ± | 0.01 |  |  |  |
| **β** | **ILO** | 0.02 | ± | 0.01 | 0.02 | ± | 0.01 |  |  |  | 0.02 | ± | 0.01 | 0.02 | ± | 0.01 | 0.02 | ± | 0.01 | *.4279* | *.8189* | *.2880* |
| (mL^-1^) | **C** | 0.02 | ± | 0.02 | 0.02 | ± | 0.01 |  |  |  | 0.02 | ± | 0.01 | 0.02 | ± | 0.01 | 0.02 | ± | 0.01 |  |  |  |
| **C** | **ILO** | 2.29 | ± | 0.49 | 1.28 | ± | 0.33 * | 2.01 | ± | 0.50 †‡ | 1.89 | ± | 0.64 † | 1.53 | ± | 0.69 * | 1.53 | ± | 0.50 * | ***<.0001*** | *.0624* | ***.0116*** |
| (mL mmHg^-1^) | **C** | 2.11 | ± | 0.93 | 1.08 | ± | 0.33 * | 1.16 | ± | 0.31 * | 1.11 | ± | 0.33 * | 1.07 | ± | 0.28 * | 1.09 | ± | 0.25 * |  |  |  |
| **Emax/Ea** | **ILO** | 1.12 | ± | 0.11 | 1.29 | ± | 0.29 |  |  |  | 1.03 | ± | 0.15 | 0.97 | ± | 0.17 | 0.93 | ± | 0.20 | ***<.0001*** | *.7329* | *.8377* |
|  | **C** | 1.11 | ± | 0.46 | 1.01 | ± | 0.31 |  |  |  | 0.97 | ± | 0.33 | 1.01 | ± | 0.29 | 0.97 | ± | 0.27 |  |  |  |

Pre-inhal. = before inhalation, n min = n minutes after inhalation of either iloprost (ILO) or control (C)

REF = RV ejection fraction; τ/RR = time constant of ventricular relaxation, corrected for the RR-interval; β = chamber stiffness constant of end-diastolic pressure volume relationship; C = PA-compliance; Emax/Ea = ratio of the slope (Emax) of the endsystolic pressure-volume relationship over effective pulmonary arterial elastance

Mean ± SD; * = P < 0.05 vs. Baseline; † = P < 0.05 vs. before inhalation; ‡ = P < 0.05 Iloprost vs. Control (corrected for multiple comparisons)

p-values of the RMANOVA are shown separately for the time-, group- and interaction- (INT, time x group) effects.
