# Supplementary material for: Effects of inhaled iloprost on right ventricular contractility, right ventriculo-vascular coupling and ventricular interdependence: a randomized placebo-controlled trial in an experimental model of acute pulmonary hypertension
Source: Crit Care. 2008 Sep 10;12(5):R113. doi: 10.1186/cc7005 (PMC2592739; doi:10.1186/cc7005)
Supplement: Additional file 5 — is a table listing the complete experimental time course of RV oxygen balance in animals subjected to acute PHT. [file cc7005-S5.doc]

**Additional data file 5:**

Parameters of Right Ventricular Oxygen Balance in Animals subjected to Acute Pulmonary Hypertension: Complete Experimental Time Course

|  |  | **Baseline** | | | **Pulmonary Hypertension** | | | | | | | | | | | | ***RMANOVA*** | | |
| --- | --- | --- | --- | --- | --- | --- | --- | --- | --- | --- | --- | --- | --- | --- | --- | --- | --- | --- | --- |
|  |  |  |  |  | **Pre-inhal.** | | | **5 min** | | | **10 min** | | | **30 min** | | | *Time* | *Group* | *INT* |
| **RCA-PP** | **ILO** | 86 | ± | 15 | 61 | ± | 22 * | 78 | ± | 18 *†‡ | 71 | ± | 18 *† | 61 | ± | 18 * | ***<.0001*** | *.0561* | ***.0009*** |
| (mmHg) | **C** | 73 | ± | 13 | 51 | ± | 9 * | 53 | ± | 12 * | 50 | ± | 13 * | 52 | ± | 9 * |  |  |  |
| **RPP** | **ILO** | 2105 | ± | 342 | 4390 | ± | 766 * | 2904 | ± | 727 *†‡ | 3361 | ± | 666 *†‡ | 4305 | ± | 1009 * | ***<.0001*** | ***.0413*** | ***.0041*** |
| (bpm · mmHg) | **C** | 2343 | ± | 816 | 4373 | ± | 997 * | 4414 | ± | 995 * | 4583 | ± | 760 * | 4882 | ± | 1062 * |  |  |  |
| **Δ HR · PVA** | **ILO** | 100 | ± | 0 | 191 | ± | 66 * | 122 | ± | 29 †‡ | 145 | ± | 44 †‡ | 177 | ± | 67 | ***<.0002*** | *n.a.* | *n.a.* |
| (%) | **C** | 100 | ± | 0 | 188 | ± | 44 * | 197 | ± | 49 * | 213 | ± | 43 * | 223 | ± | 60 * | ***<.0009*** | *n.a.* | *n.a.* |

Pre-inhal. = before inhalation, n min = n minutes after inhalation of either iloprost (ILO) or control (C)

RCA-PP = right coronary artery perfusion pressure; RPP = right ventricular rate pressure product; Δ HR · PVA = changes in the product of heart rate and pressure volume area

Mean ± SD; * = P < 0.05 vs. Baseline; † = P < 0.05 vs. before inhalation; ‡ = P < 0.05 Iloprost vs. Control (corrected for multiple comparisons)

p-values of the RMANOVA are shown separately for the time-, group- and interaction- (INT, time x group) effects.
