# Supplementary material for: Effects of inhaled iloprost on right ventricular contractility, right ventriculo-vascular coupling and ventricular interdependence: a randomized placebo-controlled trial in an experimental model of acute pulmonary hypertension
Source: Crit Care. 2008 Sep 10;12(5):R113. doi: 10.1186/cc7005 (PMC2592739; doi:10.1186/cc7005)
Supplement: Additional file 6 — is a table listing the complete experimental time course of conductance catheter derived parameters of LV function in animals subjected to acute PHT. [file cc7005-S6.doc]

**Additional data file 6:**

Conductance Catheter derived Parameters of Left Ventricular Function in Animals subjected to Acute Pulmonary Hypertension: Complete Experimental Time Course.

|  |  | **Baseline** | | | **Pulmonary Hypertension** | | | | | | | | | | | | | | | ***RMANOVA*** | | |
| --- | --- | --- | --- | --- | --- | --- | --- | --- | --- | --- | --- | --- | --- | --- | --- | --- | --- | --- | --- | --- | --- | --- |
|  |  |  |  |  | **Pre-inhal.** | | | **1 min** | | | **5 min** | | | **10 min** | | | **30 min** | | | *Time* | *Group* | *INT* |
| **Mw** | **I** | 8.29 | ± | 1.66 | 10.67 | ± | 2.45 * |  |  |  | 8.08 | ± | 2.22 | 8.11 | ± | 1.75 | 8.25 | ± | 3.40 | ***.0026*** | *.8191* | *.1223* |
| (mWatt s mL^-1^) | **C** | 7.54 | ± | 1.06 | 9.01 | ± | 1.27 * |  |  |  | 9.24 | ± | 2.69 | 8.80 | ± | 1.56 | 9.27 | ± | 1.31 |  |  |  |
| **Emax** | **ILO** | 1.10 | ± | 0.46 | 1.51 | ± | 0.74 |  |  |  | 1.20 | ± | 0.66 | 1.40 | ± | 0.80 | 1.48 | ± | 0.74 | *.1549* | *.5536* | *.2510* |
| (mmHg mL^-1^) | **C** | 1.13 | ± | 0.62 | 1.29 | ± | 0.75 |  |  |  | 1.64 | ± | 0.95 | 1.67 | ± | 0.82 | 1.72 | ± | 0.83 |  |  |  |
| **LVEF** | **ILO** | 61 | ± | 5 | 63 | ± | 12 | 56 | ± | 8 | 59 | ± | 13 | 59 | ± | 14 | 56 | ± | 12 | *.0327* | *.0964* | *.8069* |
| (%) | **C** | 57 | ± | 9 | 53 | ± | 7 | 47 | ± | 13 | 50 | ± | 5 | 50 | ± | 8 | 51 | ± | 6 |  |  |  |
| **τ/RR** | **ILO** | 0.06 | ± | 0.02 | 0.08 | ± | 0.02 | 0.07 | ± | 0.01 | 0.07 | ± | 0.01 | 0.07 | ± | 0.01 | 0.07 | ± | 0.01 | *.3723* | *.3272* | *.7082* |
| (ms) | **C** | 0.07 | ± | 0.01 | 0.07 | ± | 0.01 | 0.07 | ± | 0.01 | 0.08 | ± | 0.02 | 0.07 | ± | 0.02 | 0.07 | ± | 0.01 |  |  |  |
| **β** | **ILO** | 0.11 | ± | 0.03 | 0.11 | ± | 0.06 |  |  |  | 0.04 | ± | 0.03* | 0.07 | ± | 0.02* | 0.10 | ± | 0.03 | ***.0016*** | *.3011* | *.1366* |
| (mL^-1^) | **C** | 0.11 | ± | 0.04 | 0.13 | ± | 0.09 |  |  |  | 0.09 | ± | 0.05 | 0.11 | ± | 0.04 | 0.08 | ± | 0.03 |  |  |  |
| **C** | **ILO** | 0.72 | ± | 0.23 | 0.60 | ± | 0.20 | 0.64 | ± | 0.27 | 0.64 | ± | 0.27 | 0.64 | ± | 0.28 | 0.64 | ± | 0.22 | ***<.0001*** | *.5859* | *.0583* |
| (mL mmHg^-1^) | **C** | 0.88 | ± | 0.27 | 0.71 | ± | 0.25* | 0.67 | ± | 0.19* | 0.66 | ± | 0.21* | 0.68 | ± | 0.23* | 0.65 | ± | 0.21* |  |  |  |
| **Ea** | **ILO** | 1.87 | ± | 0.51 | 1.68 | ± | 0.44 | 1.62 | ± | 0.42 | 1.67 | ± | 0.50 | 1.75 | ± | 0.44 | 1.91 | ± | 0.45 | *.8391* | *.3880* | *.2367* |
| (mmHg mL^-1^) | **C** | 1.80 | ± | 0.46 | 1.93 | ± | 0.55 | 1.96 | ± | 0.62 | 1.95 | ± | 0.56 | 1.91 | ± | 0.54 | 1.92 | ± | 0.44 |  |  |  |
| **Emax/Ea** | **ILO** | 0.60 | ± | 0.24 | 0.92 | ± | 0.50 |  |  |  | 0.69 | ± | 0.30 | 0.73 | ± | 0.33 | 0.75 | ± | 0.36 | *.4332* | *.8118* | *.0976* |
|  | **C** | 0.62 | ± | 0.24 | 0.67 | ± | 0.31 |  |  |  | 0.86 | ± | 0.48 | 0.90 | ± | 0.48 | 0.89 | ± | 0.43 |  |  |  |

Pre-inhal. = before inhalation of iloprost, n min = n minutes after inhalation of either iloprost (ILO) or control (C)

Emax = slope of the end-systolic pressure-volume relationship (ESPVR); Mw = slope of the preload-recruitable stroke work relationship; LVEF = LV ejection fraction; τ/RR = time constant of ventricular relaxation, corrected for the RR-interval; β = chamber stiffness constant of end-diastolic pressure volume relationship; C = aortic compliance; Ea = effective arterial elastance

Mean ± SD; * = P < 0.05 vs. Baseline; † = P < 0.05 vs. before inhalation; ‡ = P < 0.05 Iloprost vs. Control (corrected for multiple comparisons)

p-values of the RMANOVA are shown separately for the time-, group- and interaction- (INT, time x group) effects.
