# Supplementary material for: Effects of inhaled iloprost on right ventricular contractility, right ventriculo-vascular coupling and ventricular interdependence: a randomized placebo-controlled trial in an experimental model of acute pulmonary hypertension
Source: Crit Care. 2008 Sep 10;12(5):R113. doi: 10.1186/cc7005 (PMC2592739; doi:10.1186/cc7005)
Supplement: Additional file 7 — is a table listing general hemodynamics in animals with and without blockade of the autonomous nervous system. [file cc7005-S7.doc]

**Additional data file 7:**

General Hemodynamics in Animals with (ANS) or without (C) Blockade of the Autonomous Nervous System

(n min = minutes after inhalation of iloprost)

|  |  |  |  |  |  |  |  |  |  |  |  |  |  |  |  |  | ***RMANOVA*** | | |
| --- | --- | --- | --- | --- | --- | --- | --- | --- | --- | --- | --- | --- | --- | --- | --- | --- | --- | --- | --- |
|  |  | **Baseline** | | | **1 min** | | | **5 min** | | | **10 min** | | | **30 min** | | | ***Time*** | ***Group*** | ***INT*** |
| **HR** | **ANS** | 103 | ± | 20 | 97 | ± | 13 | 97 | ± | 14 | 98 | ± | 15 | 95 | ± | 14 | *.7057* | *.2595* | *.2595* |
| (min^-1^) | **C** | 84 | ± | 13 | 85 | ± | 12 | 85 | ± | 13 | 84 | ± | 13 | 85 | ± | 12 |  |  |  |
| **CO** | **ANS** | 4.5 | ± | 1.0 | 4.3 | ± | 1.1 | 4.5 | ± | 1.2 | 4.4 | ± | 1.2 | 4.2 | ± | 1.3 | *.1825* | *.2606* | *.7021* |
| (L min^-1^) | **C** | 3.8 | ± | 0.6 | 3.7 | ± | 0.4 | 3.7 | ± | 0.5 | 3.5 | ± | 0.5 | 3.4 | ± | 0.6 |  |  |  |
| **SV** | **ANS** | 44 | ± | 8 | 44 | ± | 8 | 46 | ± | 8 | 45 | ± | 8 | 43 | ± | 9 | ***.0228*** | *.9320* | *.4485* |
| (mL) | **C** | 46 | ± | 8 | 45 | ± | 7 | 45 | ± | 7 | 42 | ± | 7 | 41 | ± | 8 |  |  |  |
| **MAP** | **ANS** | 97 | ± | 25 | 88 | ± | 26 * | 89 | ± | 26 * | 88 | ± | 26 * | 89 | ± | 26 * | ***.0359*** | *.6831* | ***.0170*** |
| (mmHg) | **C** | 81 | ± | 23 | 80 | ± | 23 | 86 | ± | 24 | 81 | ± | 24 | 81 | ± | 26 |  |  |  |
| **MPAP** | **ANS** | 16 | ± | 2 | 13 | ± | 3 * | 13 | ± | 3 | 14 | ± | 4 | 15 | ± | 4 | ***< .001*** | *.4775* | *.3109* |
| (mmHg) | **C** | 18 | ± | 1 | 13 | ± | 1 * | 15 | ± | 1 * | 16 | ± | 2 | 17 | ± | 2 |  |  |  |
| **LVEDP** | **ANS** | 9 | ± | 1 | 8 | ± | 1 | 9 | ± | 1 | 9 | ± | 1 | 9 | ± | 1 | *.7034* | *.2645* | *.2853* |
| (mmHg) | **C** | 9 | ± | 1 | 10 | ± | 1 | 10 | ± | 1 | 9 | ± | 1 | 10 | ± | 1 |  |  |  |
| **RVEDP** | **ANS** | 9 | ± | 2 | 10 | ± | 2 | 10 | ± | 2 | 10 | ± | 1 | 10 | ± | 2 | *.9567* | ***.0186*** | *.2207* |
| (mmHg) | **C** | 8 | ± | 1 | 7 | ± | 0 | 7 | ± | 0 | 7 | ± | 1 | 7 | ± | 1 |  |  |  |
| **SVR** | **ANS** | 1554 | ± | 247 | 1423 | ± | 149 | 1337 | ± | 222 | 1417 | ± | 259 | 1503 | ± | 241 | *.8126* | *.4234* | *.1990* |
| (dyn s cm^-5^) | **C** | 1543 | ± | 287 | 1536 | ± | 313 | 1656 | ± | 279 | 1660 | ± | 331 | 1670 | ± | 350 |  |  |  |
| **PVR** | **ANS** | 126 | ± | 43 | 85 | ± | 38 | 83 | ± | 37 | 99 | ± | 40 | 114 | ± | 45 | ***< .001*** | *.2812* | ***.0384*** |
| (dyn s cm^-5^) | **C** | 183 | ± | 40 | 72 | ± | 20 * | 104 | ± | 45 * | 156 | ± | 68 | 160 | ± | 60 |  |  |  |
| **PVR/SVR** | **ANS** | 0.09 | ± | 0.02 | 0.06 | ± | 0.02 * | 0.06 | ± | 0.03 | 0.07 | ± | 0.03 | 0.08 | ± | 0.03 | ***< .001*** | *.5402* | *.1281* |
|  | **C** | 0.12 | ± | 0.04 | 0.05 | ± | 0.01 | 0.07 | ± | 0.03 | 0.10 | ± | 0.06 | 0.10 | ± | 0.05 |  |  |  |

HR = heart rate; CO = cardiac output; SV = stroke volume; M(P)AP = mean (pulmonary) arterial pressure; L(R)VEDP = left (right) ventricular enddiastolic pressure; S(P)VR = systemic (pulmonary) vascular resistance

Mean ± SD; * = P < 0.05 vs. Baseline (corrected for multiple comparisons).

p-values of the RMANOVA are shown separately for the time-, group- and interaction- (INT, time x group) effects.
